# Effects of a Multi-Session Cognitive Training Combined With Brain Stimulation (TrainStim-Cog) on Age-Associated Cognitive Decline – Study Protocol for a Randomized Controlled Phase IIb (Monocenter) Trial

**DOI:** 10.3389/fnagi.2019.00200

**Published:** 2019-08-16

**Authors:** Daria Antonenko, Friederike Thams, Jessica Uhrich, Annika Dix, Franka Thurm, Shu-Chen Li, Ulrike Grittner, Agnes Flöel

**Affiliations:** ^1^Department of Neurology, Universitätsmedizin Greifswald, Greifswald, Germany; ^2^Chair of Lifespan Developmental Neuroscience, Faculty of Psychology, TU Dresden, Dresden, Germany; ^3^Berlin Institute of Health (BIH), Berlin, Germany; ^4^Charité – Universitätsmedizin Berlin, Corporate Member of Freie Universität Berlin, Humboldt-Universität zu Berlin, and Berlin Institute of Health, Institute of Biometry and Clinical Epidemiology, Berlin, Germany; ^5^German Centre for Neurodegenerative Diseases (DZNE) Standort Greifswald, Greifswald, Germany

**Keywords:** transcranial direct current stimulation, aging, cognitive training, working memory, decision-making, spatial learning, transfer

## Abstract

**Background:**

With increasing aging populations worldwide, developing interventions against age-associated cognitive decline is particularly important. Evidence suggests that combination of brain stimulation with cognitive training intervention may enhance training effects in terms of performance gain or transfer to untrained domains. This protocol describes a Phase IIb clinical trial that investigates the intervention effects of training combined with brain stimulation in older adults.

**Methods:**

The TrainStim-Cog study is a monocentric, randomized, single-blind, placebo-controlled intervention. The study will investigate cognitive training with concurrent anodal transcranial direct current stimulation (tDCS) over the left dorsolateral prefrontal cortex (target intervention) compared to cognitive training with sham stimulation (control intervention) over nine sessions in 3 weeks, consisting of a letter updating task, and a three-stage Markov decision-making task. Fifty-six older adults will be recruited from the general population. Baseline assessment will be performed including neuropsychological screening and performance on training tasks. Participants will be allocated to one of the two study arms using block-wise randomization stratified by age and baseline performance with a 1:1 allocation ratio. Primary outcome is performance in the letter updating task after training under anodal tDCS compared to sham stimulation. Secondary outcomes include performance changes in the decision-making task and transfer tasks, as well as brain structure and functional networks assessed by structural, and functional magnetic resonance imaging (MRI) that are acquired pre- and post-intervention.

**Significance:**

The main aim of the TrainStim-Cog study is to provide evidence for behavioral and neuronal effects of tDCS-accompanied cognitive training and to elucidate the underlying mechanisms in older adults. Our findings will contribute toward developing efficient interventions for age-associated cognitive decline.

**Trial registration:**

This trial was retrospectively registered at Clinicaltrials.gov Identifier: NCT03838211 at February 12, 2019, https://clinicaltrials.gov/ct2/show/NCT03838211.

**Protocol version:**

Based on BB 004/18 version 1.2 (May 17, 2019).

## Introduction

Given the worldwide increase of the proportion of older adults, the development of interventions against age-related cognitive declines are of great scientific and public interest (United Nations, 2015). Cognitive fitness and preserved everyday life abilities in advanced age is considered one of the most desirable conditions for individual well-being (Woods et al., 2013; Yam and Marsiske, 2013; Yam et al., 2014).

The combination of brain stimulation and multi-session cognitive training may counteract and delay the onset of age-related impairments (Mameli et al., 2014; Perceval et al., 2016). Concurrent application of transcranial direct current stimulation (tDCS) over relevant brain regions during task practice has the potential to improve task performance and induce sustained effects (Kuo and Nitsche, 2012; Mameli et al., 2014; Woods et al., 2016; Au et al., 2017; Berryhill, 2017; Antonenko et al., 2018). Studies that applied anodal tDCS over frontal brain regions during working memory practice in healthy older adults have shown maintained benefits for trained and untrained visuospatial memory abilities and everyday life-relevant tasks (Jones et al., 2015; Stephens and Berryhill, 2016). Even if immediately measurable effects are absent (Nilsson et al., 2017), this intervention holds promise to exert long-lasting benefits by increasing neural plasticity (Jones et al., 2015). Therefore, clinical trials in older adults have to assess multiple cognitive outcomes, including those relevant for activities of daily living, and cover multiple time points, including long-term follow-up-assessment.

Modulation of brain network functioning has been suggested as potential underlying mechanism of behavioral improvement through tDCS (Meinzer et al., 2012; Hunter et al., 2013; Elmasry et al., 2015). Effects of tDCS-assisted multi-session cognitive training on structural and functional brain plasticity, and their relation to changes in cognitive scores, are however, largely unknown. In a recent study, we found increased functional coupling in the default mode network in older adults that was associated with immediate episodic memory training gains (Antonenko et al., 2018). Individual microstructural properties of white matter pathways may mediate neural and behavioral plasticity induced by tDCS (Lindenberg et al., 2013; Bachtiar et al., 2018). Thus, augmented neural network functioning may result in functional effects.

However, evidence for cognitive benefits, maintenance, and transfer of practice effects to untrained abilities is still mixed with high variability among older adults and research studies (Horvath et al., 2015; Antonenko et al., 2017; Kulzow et al., 2017; Nilsson et al., 2017; Passow et al., 2017). To be able to draw firm conclusions about the efficiency of these combined interventions, well-controlled randomized clinical trials in older adults with age-associated cognitive decline are required. In addition, investigations with cognitively impaired older adults can be based on results from studies in healthy adults (Perceval et al., 2016; Summers et al., 2016).

In the TrainStim-Cog study, we will conduct a randomized clinical trial implementing a multi-session working memory training in older adults (*n* = 56). Anodal tDCS over the dorsolateral prefrontal cortex (DLPFC) will be applied during task practice in half of the participants while the other half will receive sham stimulation. Stratified randomization will assure that the two groups are comparable regarding their age and baseline performance on the trained memory updating task. Effects on performance in trained and non-trained abilities as well as on brain function and structure will be assessed at multiple time-points. The current protocol describes the design and methods implemented in the TrainStim-Cog study. This protocol was prepared in accordance with the SPIRIT guidelines (Chan et al., 2013a, b).

## Methods: Participants, Intervention, and Outcomes

### Design and Setting

**FIGURE 1:**
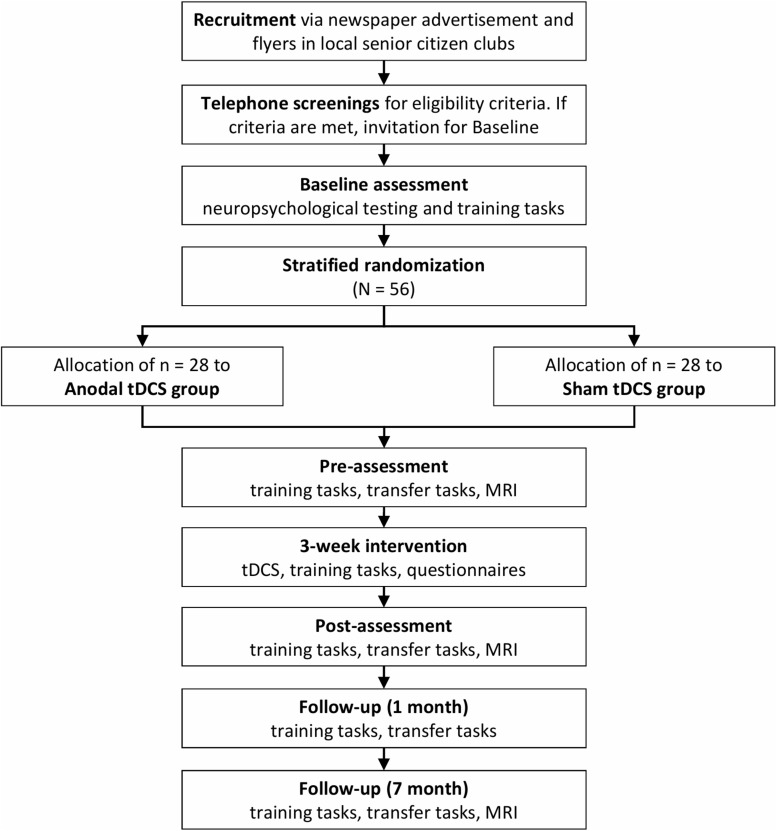
TrainStim-Cog study flowchart. tDCS, transcranial direct current stimulation; MRI, magnetic resonance imaging.

This is a monocentric, randomized, single-blind, placebo-controlled study, including a nine-session cognitive training intervention in 3 weeks, accompanied by anodal tDCS over the left DLPFC compared to sham tDCS. Subjects will participate altogether in 14 sessions with pre- and post-intervention assessments that include magnetic resonance imaging (MRI), taking place at University Medicine Greifswald. Two follow-up sessions (at one and 7 months post-training) will be performed to also assess possible maintenance effects. A flow chart of the study is shown in Figure 1.

### Eligibility Criteria

Before randomization, participants eligible for the study must meet all following criteria:

(1) Age: 65–80 years.
(2) Right-handedness.
(3) Performance in neuropsychological screening at baseline within normal range (defined as performance of each subtest within −1.5 standard deviations (SD) from the normative sample’s mean).

In case one or more of the following criteria are present at randomization, potential participants will be excluded:

(1) Mild cognitive impairment (MCI), subjective cognitive decline (SCD), or dementia (participants reporting decline in cognitive functions or performing below −1.5 SD in any neuropsychological screening subtest will be excluded).
(2) Other neurodegenerative neurological disorders; epilepsy or history of seizures; close relatives with epilepsy or history of seizures; previous stroke.
(3) Severe and untreated medical conditions that preclude participation in the training, as determined by responsible physician.
(4) History of severe alcoholism or use of drugs.
(5) Severe psychiatric disorders such as depression (if not in remission) or psychosis.
(6) Contraindication to MRI (claustrophobia, metallic implants, ferromagnetic metals in the body, disorders of thermoregulation, and pregnant women) and tDCS (cf. Antal et al., 2017).

If all eligibility criteria are met and participants provide written informed consent, they will be included in the study sample.

### Intervention

**FIGURE 2:**
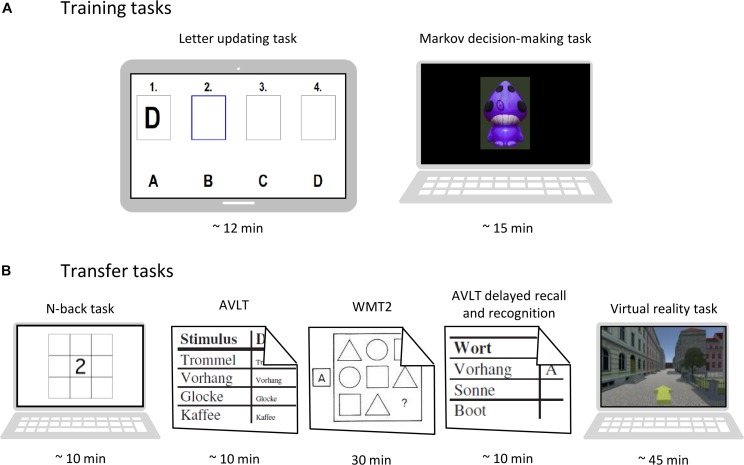
Task overview. **(A)** Training tasks performed at each session. **(B)** Transfer tasks performed at pre-, post-, and follow-up-assessments. AVLT, auditory verbal learning test (Helmstaedter et al., 2001). WMT-2, Wiener matrices test (Formann et al., 2011).

In each of the nine training sessions, participants will receive either anodal or sham tDCS while performing two cognitive training tasks, which are displayed in Figure 2A. Before starting the two training tasks, tDCS set-up will be mounted and stimulation will be started.

First, participants are presented with a letter updating task (cf. Dahlin et al., 2008) on a tablet computer, which will train updating of information stored in working memory. Lists of letters A to D (with lengths of 5, 7, 9, 11, 13, or 15 letters; three times each; total of 18 lists) will be presented in random order, one letter at a time (presentation duration: 2000 ms, ISI 500 ms). After each list, participants will be asked to recall the last four letters that were presented.

The second training task will be a three-stage Markov decision-making task (Eppinger et al., 2015; cf. Tanaka et al., 2016; Wittkuhn et al., 2018), presented on a laptop computer. Participants will be instructed to choose between two actions, i.e., pressure of left or right key, which results in an action-related reward (monetary gain or loss). Participants will have to learn to choose the optimal sequence of action to maximize overall gains and minimize overall losses. Markov probability refers to the fact that a decision at a given stage not only determines reward, but also the transition into the next stage (out of three stages) of stimulus choice decision. Therefore, choosing the optimal sequence of action will result in continuously transitioning through all three stages. The task will consist of two different learning conditions. In the *immediate reward condition*, the optimal action choice will consistently be rewarded with a gain (+€0.05). Choosing the other alternative will be punished (−€0.05), meaning that action-outcome associations will be equal for all three stages. In the *delayed reward condition*, optimal action choice will be associated with a small loss (−€0.05) in the first two stages and a large gain (+€0.25) in the third stage. Suboptimal action choice however, will be associated with a small gain (+€0.05) in the first two stages and a large loss (−€0.25) in the third stage. Thus, action-outcome associations will vary over the three stages in the delayed reward condition.

Transcranial direct current stimulation will be administered via a battery-operated stimulator (Neuroelectrics Starstim Home Research Kit, Barcelona, Spain). Direct current will be delivered with 1 mA intensity via two round saline-soaked synthetic sponge electrodes (5 cm diameter), connected to the stimulator and mounted in a neoprene head cap using the 10–20 EEG-system grid. Stimulation will consist of 20 min of continuous stimulation with ten additional seconds of ramping at the beginning and end of stimulation, respectively. The anodal electrode will be placed over left DLPFC (F3). Placement of the cathodal electrode is the contralateral supraorbital cortex (Fp2). In the sham tDCS group, the same electrode montage and ramp-time will be used, but current will only be applied for 30 s to elicit the typical tingling sensation of stimulation on the scalp and to blind participants regarding the stimulation condition (see section “Blinding”). Stimulation will be started simultaneously with the letter updating task and run during its entire length and approximately the first half of the Markov task. After every third session, participants will fill out an adverse-events questionnaire (Antal et al., 2017). Participants will be instructed to avoid excessive alcohol consumption or smoking on the day of the study, to adhere to their usual sleep duration, and to avoid drinking caffeine 90 min prior to the training sessions.

### Outcome Measures

Outcome measures for the training tasks will be acquired at each session. At pre-, post- and follow-up-assessments, additional outcome measures targeting transfer effects (see Figure 2B for an overview), will be assessed. All measures and time points of acquisition are listed in Table 1. Each outcome measure will be analyzed regarding potential differences between intervention groups (anodal vs. sham tDCS).

**TABLE 1:** TrainStim-Cog outcome measures.

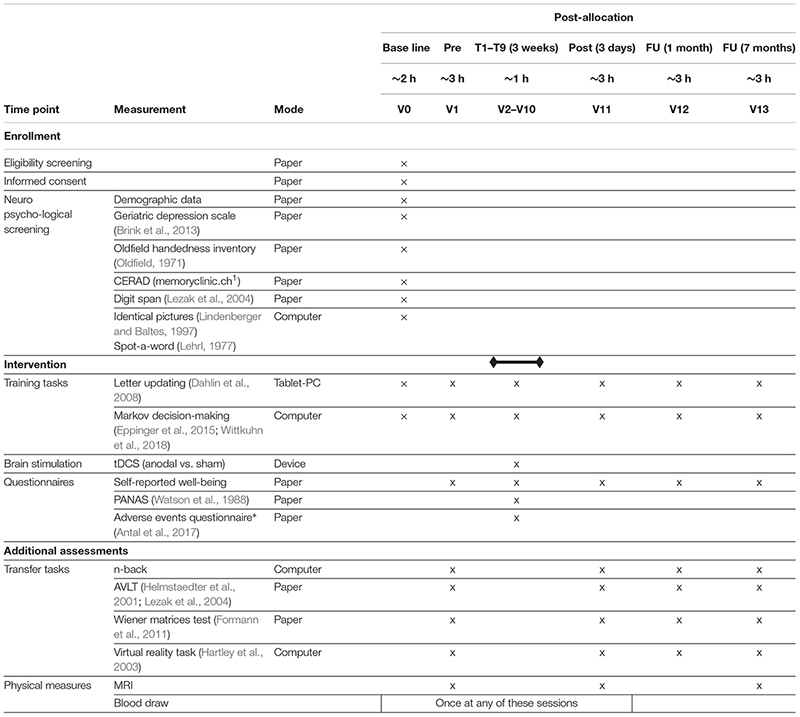

*T1–T9, training 1–9; FU, follow-up-assessment; V0–V13, visits 0–13; CERAD, the consortium to establish a registry for Alzheimer’s Disease, neuropsychological battery. PANAS, positive and negative affect schedule; AVLT, German version of the auditory verbal learning test; tDCS, transcranial direct current stimulation; MRI, magnetic resonance imaging. All measures were acquired on site, except for screening, which was done via telephone. ^∗^ assessed only at the end of each training week (V4, 7, and 10). ^1^http://www.memoryclinic.ch/de/main-navigation/neuropsychologen/cerad-plus/.*

#### Primary Outcomes

Primary outcome measure will be working memory performance at post-assessment, operationalized by number of correctly recalled lists in the letter updating task.

#### Secondary Outcomes

Secondary outcome will be performance in decision-based learning at post-assessment, as measured by proportion of optimal actions in the Markov decision-making task.

Additionally, performance at follow-up-assessments will be analyzed for the main measures of both training tasks (number of correctly recalled lists in the letter updating task and proportion of optimal actions in the Markov decision-making task). Further, online and offline effects of the intervention will be assessed for the main measures of both training tasks. Online effects will be defined as within session performance changes, whereas changes in performance from the last trial of the previous visit to the first trial of the next visit will be considered as offline effects (Reis et al., 2009). The total direct effects of the intervention will be assessed by analyzing learning curves (change from first to last training session) for both training tasks measures.

Further secondary outcomes will be assessed at pre-, post-, and follow-up-assessments and include:

(a) Transfer tasks encompassing working memory performance, as assessed by performance in a numeric n-back task (% correct, d-prime); episodic memory performance, as measured by performance in the German version of the auditory verbal learning test (Helmstaedter et al., 2001; Lezak et al., 2004) (total amount of words learned, number of recalled words at delayed recall), reasoning ability, as assessed by the Wiener matrices test (WMT-2) (Formann et al., 2011) (% correct), spatial memory ability, as measured by a virtual reality maze task (Hartley et al., 2003) (number of items found on a previous encoded route). All transfer measures will be corrected for performance at pre-assessment.
(b) Structural and functional neural correlates (assessed at pre-, post-, and 7-months follow-up-assessments), as measured by structural and functional MRI.

#### Exploratory Analyses

Exploratory analyses will be conducted for more detailed outcomes of the two training tasks (e.g., outcomes dependent on list length in the letter updating task, parameters from a drift diffusion model for the Markov decision-making task). Additionally, measures of cognitive reserve such as education, baseline cognitive ability or neuropsychological status will be analyzed for identifying potential predictors of training task performance, and responsiveness to the intervention. Genetic polymorphisms such as ApoE, COMT and BDNF, derived from analysis of blood samples and related to cognition, will be included as potential modulators of response to tDCS (Fritsch et al., 2010).

### Participant Timeline

Participants will adhere to 14 sessions with three additional MRI sessions, taking place at the University Medicine Greifswald. After completion of baseline assessment (V0), participants will successively be invited to start the training sessions (V2–V10), which will take place during three consecutive weeks on 3 days a week. Three days before and after training sessions, pre- and post-assessments (V1 and V11) will be conducted. Four weeks after post-assessment a first follow-up session (V12) will be administered; second follow-up (V13) will be 7 months after post-assessment. MRI will be measured directly before and after the training (V1 and V11) and at second follow-up (V13). An overview of all visits and outcome measures is provided in Table 1.

#### Baseline Measures

At baseline assessment (V0), participants will give written informed consent, and participate in a demographic interview. Depression screening and handedness questionnaire will be administered. Furthermore, performance in several cognitive domains will be tested (Table 1). Afterward, participants will perform the two training tasks as described above, except that at baseline, the letter updating task will consist of one practice trial with 4 lists and one actual trial with 15 lists (compared to one trial of 18 lists in the training). Baseline visit will take approximately 3 h.

#### Pre-, Post-, and Follow-Up-Assessments

All four sessions will comprise the same procedure. Initially, self-reported well-being, quality, and duration of sleep as well as potential stressors 2 h prior to the session are assessed by the investigator in a semi-structured interview. After having performed the two training tasks, participants will accomplish several transfer tasks, testing multiple memory functions, and reasoning ability. The 7-months follow-up-assessment will provide the possibility for assessing the maintenance of training and transfer effects.

### Sample Size

Based on recent studies in the field using multi-session application of anodal tDCS during cognitive training compared to training with sham tDCS (Park et al., 2014; Jones et al., 2015; Antonenko et al., 2018), we estimated an effect size of 0.85. To demonstrate an effect in the primary outcome, 46 participants (23 per group) need to be included in the analysis with an independent *t*-test using a two-sided significance level of 0.05 and a power of 80%. This conservative approach using a *t*-test was chosen, even though we intend to analyze the primary outcome conducting analysis of covariance (ANCOVA) models (Borm et al., 2007). Assuming a drop-out rate of about 20% due to a high number of planned visits and considerably high demands put upon participants (e.g., performing challenging memory tasks and attending three sessions of 45 min MRI scans), 28 participants should be included in each tDCS group. Sample size estimation was conducted using G^∗^Power 3.1 (Faul et al., 2007).

### Recruitment

Participants will be recruited through advertisements in the local newspapers and distribution of flyers in local senior citizen clubs. Telephone screenings will be conducted with all potential participants and study information will be provided. Eligible candidates will be invited for baseline assessment. Following baseline assessment (V0) participants will be included if neuropsychological testing is unobtrusive.

## Methods: Assignment of Interventions

We will first complete recruitment and baseline measurement of all subjects before allocating participants to the groups and starting the training sessions. Allocation will be performed by a researcher not involved in baseline assessments. Participants will be randomly allocated in 1:1 ratio to the experimental groups (anodal vs. sham tDCS), using age and initial performance in the letter updating task as strata. First, all participants that successfully completed telephone screening and baseline assessments will be divided into four groups by median split: (1) age ≤ median and LU performance ≤ median, (2) age ≤ median and LU performance > median, (3) age > median and LU performance ≤ median, and (4) age > median and LU performance > median. Second, equal numbers of participants of each group will be randomly assigned to anodal and sham tDCS group, respectively, using the blockrand package in R^1^ ^,2^ ^,3^. Allocation concealment will be ensured, since decision about in- or exclusion will already be made, before allocating participants to the groups.

### Blinding

This is a single-blind trial, participants will be blind to the stimulation condition. In the sham tDCS group, current will be applied for 30 s to elicit the typical tingling sensation of stimulation on the scalp and to blind participants regarding the stimulation condition. Previous research showed that sham tDCS is a safe and valid method of blinding participants (Schlaug and Renga, 2008; Floel and Cohen, 2010). After the last training session, participants will be asked to state whether they believed they received anodal or sham tDCS. Note that in our single-blind design, implicit bias of the investigators during data collection cannot be completely excluded. However, to minimize bias in the analysis, data will be entered electronically, and will be analyzed, by a member of the research team blinded to the stimulation condition, according to the statistical analysis plan.

## Methods: Data Collection, Management, and Analysis

### Data Collection Methods

Neuropsychological, behavioral and MRI data and blood samples will be collected from each participant. Study assessors will be thoroughly trained in administering the assessments. Time points of data collection are shown in Table 1.

#### Neuropsychological and Behavioral Assessment

Neuropsychological tests at baseline visit (V0) comprise paper-pencil as well as computer-based assessments. Geriatric Depression Scale (Brink et al., 2013) and the Edinburgh Handedness Inventory (Oldfield, 1971) will be administered. Performance in several cognitive domains will be tested with CERAD-Plus test battery^4^ (Morris et al., 1989), digit span test (Lezak et al., 2004), identical-pictures task (Lindenberger and Baltes, 1997), and spot-a-word task (Lehrl, 1977).

Training- and transfer tasks include paper-pencil and computer-based assessments. Detailed description of the two training tasks is provided in the interventions section. In pre-, post- and follow-up sessions (V1, V11–V13), transfer tasks will be administered: Participants will perform a numeric n-back task (1 and 2 back) and the German version of the auditory verbal learning test (Helmstaedter et al., 2001; Lezak et al., 2004). In the 30 min interval to assess long-term memory, participants will perform the Wiener matrices test (WMT-2) (Formann et al., 2011). Then, a virtual reality navigation task will be presented (Hartley et al., 2003). Here, during encoding, participants will be instructed to memorize a route with several targets (e.g., butcher, doctor’s office, and grocery store); during subsequent recall, the participants will be asked to navigate the shortest route to given targets.

#### Magnetic Resonance Imaging

**FIGURE 3:**
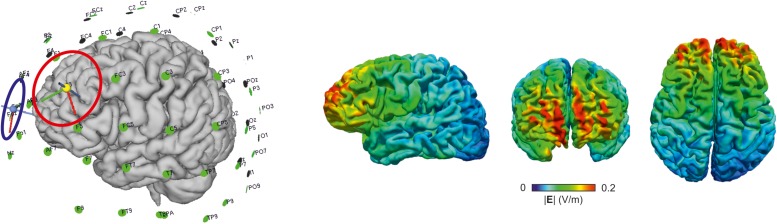
Positioning of the electrodes (anode, red; cathode, blue) and electric field distribution derived from computation modeling using SimNIBS [simnibs.org, (Windhoff et al., 2013; Thielscher et al., 2015)], for one example participant (f, 69 years).

Magnetic resonance imaging will be assessed at the Baltic Imaging Center (Center for Diagnostic Radiology and Neuroradiology, Universitätsmedizin Greifswald) with a 3 Tesla scanner (Siemens Verio) using a 32-channel head coil, 1 day prior to and 2 days as well as 7 months after training (see Table 2 for neuroimaging data acquisition parameters). A T1-weighted 3D sequence, a 3D FLAIR, a diffusion tensor imaging (DTI) and a resting-state fMRI sequence will be assessed. At the end of the MRI assessment, additional T1-and T2-weighted structural images will be acquired with parameters optimized for computational modeling to calculate electric field distributions^5^ [(Windhoff et al., 2013; Thielscher et al., 2015), see Figure 3 for sample modeling analysis]. Detailed information about MRI sequences is provided in Table 2. Pipelines from MATLAB-based toolboxes such as SPM (Wellcome Department of Imaging Neuroscience, London, United Kingdom^6^), CONN toolbox^7^ (Whitfield-Gabrieli and Nieto-Castanon, 2012), or FSL (Analysis Group, FMRIB, Oxford, United Kingdom^8^) (Jenkinson et al., 2012) and Freesurfer^9^ will be used for analysis of structural and functional MRI data. To assess volume of cortical and subcortical gray matter, structural segmentation will be performed on T1-weighted scans (Dahlin et al., 2008; Filmer et al., 2019). White matter microstructure in main white matter tracts will be extracted from diffusion-weighted images using common tractography methods (Charlton et al., 2010; Metzler-Baddeley et al., 2011, 2017, Le Bihan and Johansen-Berg, 2012). Functional resting-state fMRI scans will be used to assess functional connectivity within and between large-scale networks that mediate cognitive functions (Darki and Klingberg, 2015; Antonenko et al., 2018, 2019a).

**TABLE 2:** Neuroimaging data acquisition parameters.

**Sequence**	**Main parameters**
Resting-state fMRI	TR = 2000 ms, TE = 30 ms, FOV 192 × 192 mm^2^, 34 slices, 176 volumes, descending acquisition, 3.0 × 3.0 × 3.0 mm^3^, flip angle 90°
T1 MPRAGE	TR = 2300 ms, TE = 2.96 ms, TI = 900 ms, 192 slices, 1.0 × 1.0 × 1.0 mm^3^, flip angle 9°
DTI	TR = 11100 ms, TE = 107 ms, 70 slices, 1.8 × 1.8 × 2.0 mm^3^, 64 directions (*b* = 1000 s/mm^2^)
FLAIR	TR = 5000 ms, TE = 388 ms, 160 slices, 1.0 × 1.0 × 1.0 mm^3^
T1w	TR = 1690 ms, TE = 2.52 ms, TI = 900 ms, 176 slices, 1.0 × 1.0 × 1.0 mm^3^, flip angle 9°, using selective water excitation for fat suppression
T2w	TR = 12770 ms, TE = 86.0 ms, 96 slices, 1.0 × 1.0 × 1.0 mm^3^, flip angle 111°

*TR, repetition time; TE, echo time; TI, inversion time; FOV, field of view; fMRI, functional magnetic resonance imaging; DTI, diffusion tensor imaging; FLAIR, fluid attenuated inversion recovery; MPRAGE, magnetization prepared rapid acquisition gradient echo.*

#### Blood Draw

At one of the sessions, a blood sample for conducting genetic analyses will be collected, preprocessed, and stored at the Neuroimmunology Lab of University Medicine Greifswald, using cryo-preservation method. Genetic polymorphisms relevant for learning (such as ApoE, COMT, and BDNF) (Antal et al., 2010; Freitas et al., 2011; Witte et al., 2012; Plewnia et al., 2013) will be analyzed at the Department of Psychiatry, Psychotherapy and Psychosomatic, University Medicine, Halle/Saale, Germany.

#### Retention and Adherence

To maximize retention throughout the entire study period, participants will be provided with information about their appointments via telephone, and e-mail. A few days prior to starting the 3-weeks training period, participants will be contacted via telephone as a reminder of the upcoming appointments, and to clarify potential open questions. On pre-assessment, they will receive a printout of all study sessions. Additionally, time and date of the next session will be discussed at each visit. Participants will be encouraged to leave a message on the study site’s 24/7 answering machine, if they have any concerns about not being able to attend a visit or wanting to change the time. They will then be contacted to discuss alternative scheduling. At the end of the study, participants receive a reasonable financial reimbursement (approximately 10 € per hour), the results of their neuropsychological screening, and structural MRI images on a compact disc. If complete adherence to the protocol is not possible, any effort to collect as much data as feasible from the participant will be made.

### Data Management and Monitoring

All data collected will be pseudonymized. Files containing personal data of the participants will be saved with a password, solely known by staff involved in the project. Data acquired on paper will be entered electronically by research staff. To prevent erroneous entries, data will be entered by one person, and double-checked by another. Progress of data entry and checking procedures will be documented. Folders containing forms and questionnaires of each subject will be stored securely and sorted by participant ID number for easy access at each stage of the study. Forms containing names and personal data of participants will be stored separately in a lockable cabinet. Digital data, such as output files from computer-based tasks, will be stored on a secure file server directly after acquisition. Protocols of the tDCS setup of each participant and session will also be stored on the file server. MRI data will be pseudonymized before analysis. Following good scientific practice, data will be stored for at least 10 years.

### Adverse Events Monitoring

Potential adverse events (AEs) occurring throughout the course of the study will be monitored via an adverse events questionnaire (Antal et al., 2017), administered after each third stimulation session. We will refrain from administering the AEs-questionnaire at each stimulation session, since this might unnecessarily draw the participant’s attention to minor sensations during stimulation, and therefore serve as an unintentional distractor from the tasks. Generally, the risk of health damage due to anodal tDCS can be expected to be minimal. Known AEs with the study parameters (20 min, 1 mA) are tingling at the electrode sites, skin reddening under the electrode and, less frequently, a mild headache (Antal et al., 2017). Study assessors will be instructed to monitor AEs and serious AEs (SAEs) throughout the trial and document all detected AEs and SAEs. Participants will be informed at baseline visit about all possible risks and can withdraw consent at any time without providing reasons. In case an SAE occurs, the study physician will first make an assessment as to whether a causal relationship with the intervention is considered possible. If more than three of the enrolled participants suffer from SAEs that are likely to be associated with the intervention (as assessed by the study physician), the trial will be discontinued.

### Statistical Methods

Statistical analyses of the primary and secondary outcome measures will be reported in detail in the statistical analysis plan, to be written and registered before data analyzes. Data from all participants included at randomization will be analyzed using intention to treat analyses (ITT). In case of missing values and under the assumption of missing at random we will use multiple imputation methods to estimate treatment effects. Additionally, a “per protocol” analysis will be conducted as sensitivity analysis, including only those participants, who completed all visits, and finished post-assessment. Focusing on the primary outcome, we will conduct an ANCOVA with the post-assessment working memory score (number of correctly recalled lists in the letter updating task) as dependent variable, stimulation group (anodal, sham) as factor, and working memory performance at pre-assessment as well as age as covariates. Secondary outcomes will be analyzed using similar statistical methods. For instance, an ANCOVA with performance in the Markov decision-making task at post-assessment (proportion of optimal actions) as dependent variable, stimulation group (anodal, sham) as factor, and performance in the Markov decision-making task at pre-assessment as well as age as covariates will be conducted. We will furthermore analyze secondary outcome measures and their interactions, using linear mixed models with time-point (e.g., training days 1–9) as within-subject factor and stimulation group (anodal, sham) as between-subject factor. Changes in structural and functional neural parameters will be analyzed on whole-brain level, using general linear models, implemented in the analysis software. To assess brain-behavior associations, correlations between behavioral and neuronal parameters, will be calculated. In case of violation of requirements for parametric methods, appropriate non-parametric tests will be conducted. Data analysis will be conducted using IBM SPSS Statistics for Windows (IBM Corp., Armonk, NY, United States), MatLab (The Mathworks Inc., 2016), and R software^10^.

## Ethics and Dissemination

The study was approved by the ethics committee of the University Medicine Greifswald and will be conducted in accordance with the Helsinki Declaration. All data collected will by pseudonymized. Imaging data will be made publicly available to the general academic community at https://openneuro.org. Results of the study will be made accessible to scientific researchers and health care professionals via publications in peer reviewed journals and presentations at national and international conferences. Furthermore, the scientific and lay public can access the study results on the ClinicalTrials.gov website (Identifier: NCT03838211).

## Discussion

This randomized controlled trial will determine the impact of a multi-session memory training combined with anodal tDCS on trained and untrained functions as well as functional and structural neural parameters in cognitively intact older adults. In the target group, anodal tDCS (1 mA, 20 min) will be applied over the DLPFC (with the cathode over the contralateral supraorbital cortex) during task practice while the control group will receive sham tDCS (1 mA, 30 s). Findings of this study will contribute to the understanding of immediate and delayed behavioral and neural effects, as well as the underlying mechanisms of tDCS-plus-training effects in the aged brain.

Older adults constitute the target group of this trial as we aim to exert beneficial effects on age-related cognitive decline. We opted to elucidate the effects and underlying mechanisms in a population that serves as baseline for future trials targeting cognitively impaired older patients, for example MCI or dementia due to Alzheimer’s disease (Perceval et al., 2016).

All participants will undergo extensive baseline assessment to obtain detailed participant characteristics and to assure cognitive functioning within age- and education-related norms. Stratified randomization will be performed to match anodal and sham groups for age and baseline performance in the training task. We argue that this is important to exclude chance age and baseline differences that impact tDCS-induced modulation and hamper the interpretation of effects (Martin et al., 2013; Antonenko et al., 2017, 2018). Furthermore, as tDCS may potentially exert long-term effects, even in the absence of immediate training gains, we will invite participants to two follow-up sessions one and 7 months after training (Jones et al., 2015; Berryhill, 2017).

We designed a cognitive training with two cognitive tasks that are mediated by the prefrontal cortex, vulnerable to the effects of aging, and well suited to examine effects of practice over multiple sessions in older adults (Dahlin et al., 2008; Eppinger et al., 2015; Wittkuhn et al., 2018). The primary task (letter updating) has shown good applicability and training-induced plasticity in older adults (Dahlin et al., 2008). To study near and far transfer of training, we chose tasks which measure working and episodic memory, reasoning and visuospatial abilities. Altogether, we aimed to design a comprehensive assessment of multiple task domains at multiple time points avoiding floor or ceiling effects, which is also not too long or too exhausting for older participants. This multimodal testing, including paper-pencil, computer- and tablet-based tasks will not only provide reliable data on training and transfer effects, but also be attractive to the participants, assuring their compliance, and motivation. Stimulation parameters were chosen accordingly to induce plasticity in the prefrontal cortex and modulate performance in training tasks, as shown in previous studies of our group, and others (Martin et al., 2013; Meinzer et al., 2013; Wittkuhn et al., 2018).

Multimodal imaging will allow us to examine neural effects through several neural interdependent but also discrete parameters. Resting-state fMRI will allow quantification of functional connectivity in cortical and subcortical networks mediating memory functions such as the fronto-parietal, default mode, and salience network. DTI will allow individual fiber tractography of white matter pathways within these networks and their macro- and microstructural properties. T1-weighted images will allow cortical and subcortical segmentation and volumentry and FLAIR images the identification of white matter hyperintensities. These sequences will determine predictors of response on an individual level, but will also be examined at multiple time points (pre-, post-, and follow-up sessions) in order to scrutinize intervention-induced modulation. In addition, T1- and T2-weighted structural sequences will be optimized for the purpose of computational modeling in order to allow accurate simulations of the current flow on an individual basis (Antonenko et al., in press). Blood samples will be collected in order to examine if individual responsiveness to tDCS is modulated by genetic polymorphisms that have previously been shown to modulate neural plasticity and learning ability in several domains (Antal et al., 2010; Freitas et al., 2011; Witte et al., 2012; Plewnia et al., 2013; Stephens et al., 2017). Assessment of daily health conditions, potential pre-session stressors, mood and perceived tDCS-related adverse events will complete the assessment of performance and help to identify potential sources of variability.

Taken together, our Phase IIb clinical trial will provide comprehensive evidence for the effects of a tDCS-plus-training approach in age-associated cognitive decline and thus contribute to the understanding of plasticity-inducing interventions in aging, and informing the development of efficient interventions in the future.

## Trial Status

Recruitment of participants started May 2018. Last follow-up is expected for March 2020.

## Ethics Statement

The studies involving human participants were reviewed and approved by the ethics committee of University medicine Greifswald. The participants provided their written informed consent to participate in this study.

## Author Contributions

DA, S-CL, and AF designed the study and wrote the grant applications. DA, AD, and FraT developed and programmed the cognitive tasks. DA and AF acquired the ethical approval. DA, FriT, and JU prepared the information sheets and study materials. FriT and JU will acquire the data. DA, FriT, and UG will analyze the data. DA, FriT, and AF wrote the study protocol. All authors commented and approved the final version of the manuscript.

## Conflict of Interest Statement

The authors declare that the research was conducted in the absence of any commercial or financial relationships that could be construed as a potential conflict of interest.

## Footnotes

1 http://www.R-project.org

2 http://www.rstudio.com

3 https://CRAN.R-project.org/package=blockrand

4 http://www.memoryclinic.ch/de/main-navigation/neuropsychologen/cerad-plus/

5 http://simnibs.org

6 www.fil.ion.ucl.ac.uk/spm/

7 www.nitrc.org/projects/conn

8 http://fsl.fmrib.ox.ac.uk/fsl/fslwiki/

9 http://surfer.nmr.mgh.harvard.edu/

10 https://www.R-project.org
